# Body composition and bone health outcomes across positional groups in Netball Super League (NSL) senior and under-21 players; a multi-year cohort study

**DOI:** 10.17159/2078-516X/2025/v37i1a22881

**Published:** 2025-12-15

**Authors:** S Whitehead, S Chantler, L Mackay, B Jones, O Heyward, N Costello, DC Janse Van Rensburg, M Alexander, J Parmley, M Barlow

**Affiliations:** 1 Carnegie School of Sport, Leeds Beckett University, Leeds, United Kingdom; 2 Leeds Rhinos Netball, Leeds, United Kingdom; 3 England Netball, Loughborough, United Kingdom; 4 England Performance Unit, Rugby Football League, Manchester, United Kingdom; 5 Division of Physiological Sciences, Department of Human Biology, Faculty of Health Sciences, the University of Cape Town, Cape Town, South Africa; 6 School of Behavioural and Health Sciences, Faculty of Health Sciences, Australian Catholic University, Brisbane, QLD, Australia; 7 Premiership Rugby, London, United Kingdom; 8 Rugby Football Union, Twickenham, United Kingdom; 9 Section Sports Medicine and SEMLI, Faculty of Health Sciences, University of Pretoria, Pretoria, South Africa; 10 Medical Advisory Panel, World Netball, Manchester, United Kingdom; 11 Department of Health Professions, Institute of Sport, Manchester Metropolitan University, Manchester, United Kingdom

**Keywords:** Netball, female, body composition, bone health, physical

## Abstract

**Background:**

Body composition and bone health are important for netball from a performance and health perspective (e.g., bone stress injury), given the typical characteristics of players and demands of the game.

**Objectives:**

The objectives of this study are to quantify and compare the positional group-specific body composition and site-specific bone health outcomes of netball players and to establish within-season changes in these variables.

**Methods:**

Forty-seven female netball players (senior: n=23, under-21: n=24) from one Netball Super League (NSL) franchise participated across three seasons (2021–2023). Dual-energy X-ray absorptiometry (DEXA) scans were conducted four times per season. Total body, anteroposterior lumbar spine and total hip scans were performed. General and generalised linear mixed models were used to compare positional groups and age groups, and to investigate within-season changes.

**Results:**

Goal circle netball players had greater total mass and bone mass than midcourt netball players at both levels (*p*<0.05, effect size: *moderate* to *very large*), but not when scaled for height. Senior players had greater lean mass, bone mass, total bone mineral density and bone mineral content than under-21 players (*p*<0.05, effect size: *moderate* to *very large*). No group-level significant changes were observed across a playing season, but individual trends varied.

**Conclusion:**

These findings highlight the importance of continued physical development in the under-21 squad before progressing to a senior squad, as well as the need for individualised approaches to nutritional and training interventions that support physical development, addressing positional requirements and developmental stages. Future research should explore longitudinal body composition trajectories across career phases and multiple teams to refine normative benchmarks.

Body composition and bone health are crucial for netball from both performance and health perspectives, given the typical characteristics of netball players (e.g., low body mass, female) and the physical demands of the game (e.g., high biomechanical load).^[1]^ Performance staff, medical professionals, and coaches play a key role in managing body composition, yet limited comparative data on body composition in netball are available.^[1]^ Netball has high energy demands, peaking at 16.60 ± 2.75 MJ·day^−1^ on match days, with individual and positional variability present.^[2]^ Concerns exist in the Netball Super League (NSL; the United Kingdom’s elite competition) regarding players’ increased risk of relative energy deficiency in sport (RED-S), due to a lack of awareness of netball-specific energy demands among players, coaches, and medical staff, compounded by body image concerns and fuelling challenges.^[3]^ Furthermore, in South Africa, top female student players have been reported to have sub-optimal energy intakes.^[4]^ A greater understanding of the sport, including positional, body composition, and bone health, is required to support nutritional management in netball.

Monitoring bone health outcomes is important for netball players for short-term (i.e., bone stress injuries) and long-term (i.e., osteoporosis) health.^[5]^ The skeletal loading in netball has been linked to higher bone mineral density (BMD) compared to controls.^[6]^ Using Dual-energy X-ray absorptiometry (DEXA), research and practice apply general population normative data (i.e., z-scores) to interpret the BMD of athletes across sports. Whilst guidance suggests considering higher thresholds for athletes in weight-bearing sports^[7]^, this approach may not be sensitive enough in identifying impaired bone health, and the use of sport-specific z-scores has been proposed.^[8]^ The whole-body BMD of netball players has been reported^[6,9,10]^ with the highest values recorded in professional netball players.^[9]^ However, at the elite level, current research reports whole-body BMD only, without providing site-specific measurements (e.g., spine or hip). Site-specific bone health outcomes are important to consider, given that they are more sensitive to skeletal loading (e.g., running and jumping).^[11]^ Within netball, the physical movement characteristics vary between playing positions due to the court restrictions.^[12,13]^ Previous research has identified differences in body mass, lean mass and fat mass observed between positional groups, but no differences in whole-body BMD.^[9]^ However, the differences in movement characteristics between the positions could result in differences in skeletal loading and site-specific bone outcomes. Therefore, further research, including site-specific measurements, is required to investigate bone health outcomes in netball players across different populations, supporting the sport-specific interpretation of these findings.

Netball players encounter various training stimuli throughout a competitive season to develop physical qualities that prepare them for the demands of competition.^[9,12]^ Research investigating longitudinal changes in body composition in female team sports, and specifically netball, is limited. Hogarth et al.^[9]^ found that across a netball pre-season, players’ lean mass increased and fat mass decreased, with maintenance of both fat and lean mass across the competition (i.e., in-season) period. However, this is from one competition environment, the Suncorp Super Netball (SSN) in Australia. Four elite competitions exist worldwide (SSN, ANZ Premiership [New Zealand], NSL, Telkom Netball League [South Africa]), but differences exist between their training and competition environments (e.g., semi-professional *vs.* professional, facilities, staff provision). Therefore, further research is needed across the different competitions and their pathways. The mechanical loading in netball, resulting from frequent acceleration, deceleration, and changes of direction ^[13],^ could lead to small changes in bone characteristics across a season, as seen in other team sports (e.g., soccer).^[14]^ Whilst Hogarth et al.^[9]^ found a significant effect of time point on whole-body BMD in the SSN team, changes were *negligible*. Therefore, further research that utilises site-specific BMD measurements is warranted. Additionally, given the likely different training focus and bone accrual of youth development athletes,^[15]^ longitudinal research across different levels of competition would support the development of appropriate training and nutritional strategies.

Therefore, this study aims to: (1) quantify and compare the positional group-specific body composition and site-specific bone health outcomes of senior and under-21 NSL players; and (2) examine within-season changes in these outcomes at each playing level. This will enable more informed, individualised management that enhances player development, performance, and health.

## Methods

### Participants

Forty-seven female netball players (senior: n=23, age: 25±4 years; under-21: n=24, age: 19±1 years) from one NSL franchise volunteered to participate in this study. Senior NSL teams are the top of the domestic playing pathway in the United Kingdom. The senior teams in the NSL are open age, with a minimum age of 16 years old. At the time of data collection, the under-21 team was the top level of the NSL playing pathway, acting as the feeder competition to the NSL. The players were classified by playing positional groups: midcourt (centre, wing attack, wing defence) and goal circle (goal shooter, goal keeper, goal attack, goal defence).^[9]^ The study received ethics approval from Leeds Beckett University (102103). All participants provided written informed consent before data collection.

### Design

This multi-year cohort study (2021–2023) examined withinseason changes in body composition and bone outcomes. Across three seasons, the range of observations per player was 1 to 9 (median [interquartile range]: 3 [3]) (Supplementary Figure 1). DEXA scans were conducted four times per season, approximately every three months. Scans took place in October (time-point one [T1], start of pre-season), January (time-point two [T2], end of pre-season for senior, early in-season for under-21s), April (time-point three [T3], mid-season), and June (time-point four [T4], end of season). Table 1 provides an overview of training across the study period (T1–T4).

### DEXA procedures

Participants were assessed in lightweight clothing, barefoot, and without jewellery. Stature was measured using a free-standing stadiometer (SECA, Birmingham, UK) to the nearest 0.1 cm, and body mass was recorded using calibrated electronic scales (SECA, Birmingham, UK) to the nearest 0.1 kg.^[16]^ All scans were conducted in a euhydrated state using a fan-beam GE Lunar iDEXA (EnCore software version 18.0, GE Medical Systems, Hatfield, UK) following manufacturer and International Society Clinical Densitometry guidelines.^[17]^ Total body-, anteroposterior lumbar spine- (L1–L4), and total hip scans were performed, with BMD and bone mineral content (BMC) variables provided, as per guidelines. z-scores for BMD were sourced from the relevant database according to manufacturing standards. In-vivo precision (coefficient of variation [CV]) for the DEXA measurements for the Leeds Beckett DEXA unit is 0.82% for lumbar spine BMD, 0.98% for total hip. Total Body Composition precision (CV) measurements are 0.99% for fat, fat mass 0.98%, lean mass 0.42%. Fat, lean, and bone mass were scaled for height (kg·m^2^) to account for positional differences in stature, enabling player comparisons^.[9]^

### Statistical analysis

General and generalised linear mixed models were employed to quantify and compare body composition and bone health outcomes between positional groups and playing levels, and to investigate within-season changes. General linear mixed models were applied to all variables except total body fat percentage, which was analysed using a generalised linear mixed model with a beta distribution.^[19]^ One model was compared across levels and positional group differences, with fixed effects for level, positional group, and their interaction. Random effects for player identification and season nested within player identification accounted for repeated measures and variability between players and within players across seasons. A second model examined within-season changes, with time point, level, and their interaction as fixed effects, while player identification and season were treated as random effects. Participants were required to have at least three observations in the same season to be included in the within-season changes analysis; thus, 22 participants were excluded from the analysis (Supplementary Figure 1).

Results are presented as estimated means (95% confidence interval [CI]), with significance set at *p*<0.05. The effect sizes (ES) were classified as follows: *trivial* (<0.2), *small* (0.2–0.59), *moderate* (0.6–1.19), *large* (1.2–1.99), *very large* (2.0–4.0), or *extremely large* (>4.0). Odds ratios were used for fat percentage due to its beta distribution. Statistical analyses were conducted using R (v4.2.2, R Foundation for Statistical Computing, Vienna, Austria).

## Results

### Comparison between senior and under-21 positional groups

**Fig. 1:**
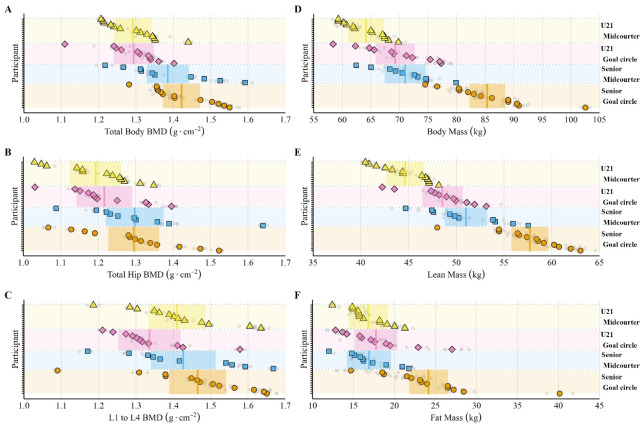
Individual participant bone outcome (A = total bone mineral density [BMD], B = total hip BMD, C = lumbar spine BMD) and body composition (D = body mass, E = lean mass, F = fat mass) for senior and under-21 (U21) goal circle and midcourters. Each symbol represents an individual player mean, with the grey dot representing individual observations for the player. Estimated mean (solid line) and 95% confidence intervals (coloured box) overlay individual values.

Body composition and bone health outcomes were compared between senior and under-21 netball players, stratified by positional group. Estimated means and 95% CI are presented in Table 2, while *p*-values and effect sizes for positional group and senior *vs.* under-21 comparisons are shown in Supplementary Table 1. Positional and age group comparisons of body composition (body mass, lean mass, and fat mass) and bone (total, spine, and hip) outcomes are presented in Figure 1, with individual player means and raw values overlaid, illustrating between-individual variability.

Senior goal circle netball players were significantly taller and presented higher body mass than senior midcourters (*p*<0.01) and under-21 goal circle players (*p*<0.01). Senior midcourters were also significantly taller and heavier than under-21 midcourters (*p*<0.01). Senior goal circle players exhibited the absolute highest fat mass, significantly exceeding both senior midcourters (*p*<0.01, *very large*) and under-21 goal circle players (*p*<0.01, *large*). Whilst there was no difference in fat mass percentage between senior and under-21 players (*p*>0.05). Absolute lean mass was greatest in senior players for both positional groups (*p*<0.01) and greatest for goal circle players in the seniors and under-21s (*p*<0.01). When scaled for height, positional differences diminished for lean mass (*p*>0.05), but differences in lean and fat mass remained between the senior and under-21s (*p*<0.01).

Total bone mass was highest in senior netball players, with no significant difference in bone mass between positions within the under-21s (*p*=0.05). Only senior goal circle players had significantly greater bone mass compared to under-21s when scaled for height (*p*<0.01, *large*). Senior netball players had a greater total BMD and BMC than under-21s (*p*<0.05), but the only positional differences were observed in BMC for senior netball players (*p*<0.01). Site-specific analysis revealed positional differences in total hip BMC at the senior level. In contrast, the total hip BMC, lumbar spine BMD, BMC, and z-score were all significantly greater for senior goal circle players compared to under-21 goal circle players (*p*<0.05).

### Within-season changes

**Fig. 2:**
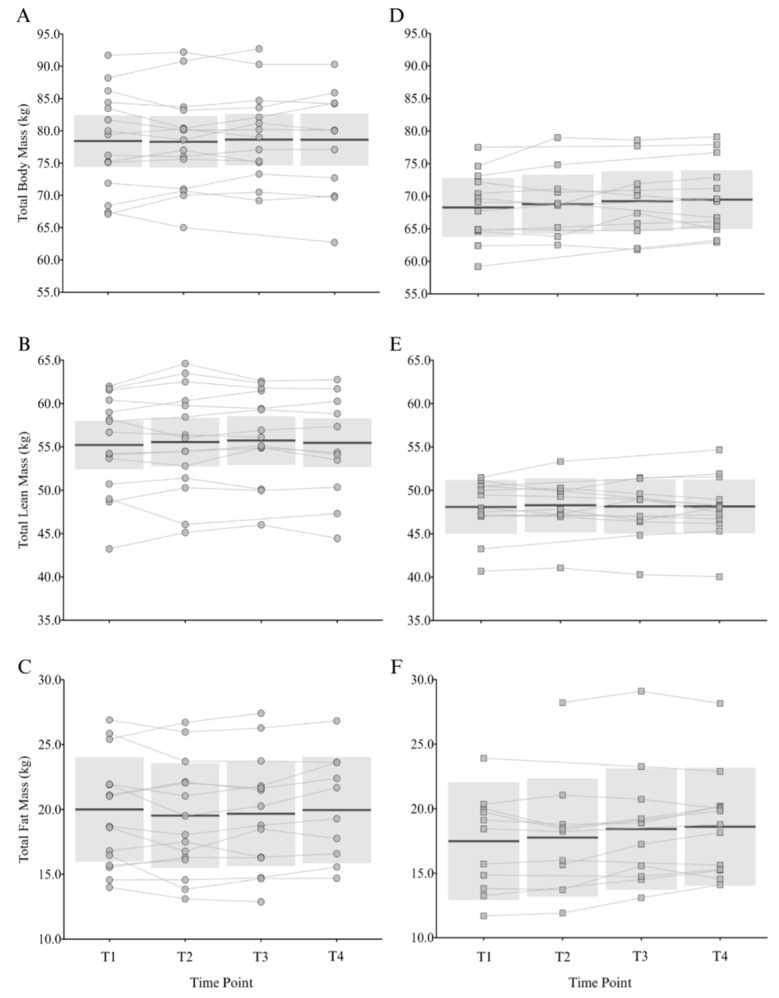
Individual and estimated mean (95% confidence interval) changes in total body mass, lean mass and fat mass across time points within a season for senior (A–C, circle) and under-21 (D–F, square) Netball Super League squad. Each line represents raw data for an individual player across time points in one season (i.e., players in multiple seasons have a line per season representing the change within each season). Time-point one (T1) - beginning of pre-season; time-point two (T2) - end of pre-season; time-point three (T3) - mid-season; time-point four (T4) - end of season.

**Fig. 3:**
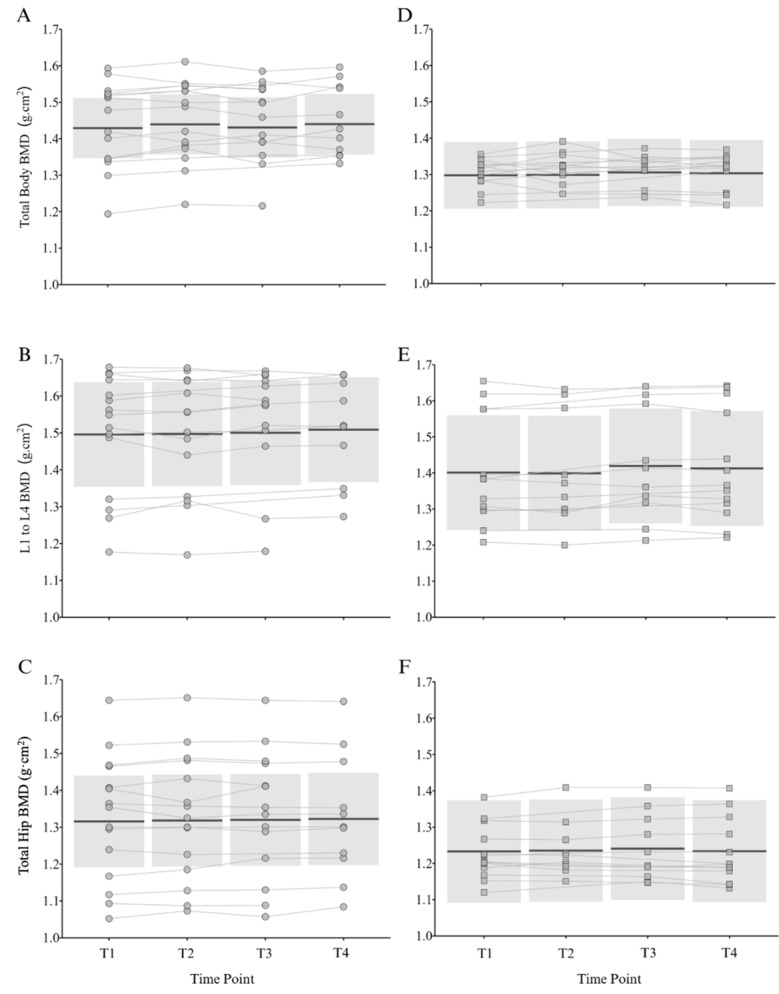
Individual and estimated mean (95% confidence interval) changes in total body, spine (L1–L4) and total hip bone mineral density (BMD) across time points within a season for senior (A–C, circle) and under-21 (D–F, square) Netball Super League squad. Each line represents raw data for an individual player across time points in one season (i.e., players in multiple seasons have a line per season representing the change within each season). Time-point one (T1) - beginning of pre-season; time-point two (T2) - end of pre-season; time-point three (T3) - mid-season; time-point four (T4) - end of season.

Figures 2 and 3 show the group level within-season changes in body composition and BMD, respectively. Individual raw values are displayed alongside the group-level comparisons to show the variability in individual responses over time. There were no significant effects of time on any of the body composition or bone outcome variables for senior or under-21 netball players.

## Discussion

This study quantified and compared the body composition and site-specific bone health outcomes of senior and under-21 elite female netball players at the positional group level, examining within-season changes. Differences were evident between age groups and positional groups, with goal circle players exhibiting greater lean mass, fat mass, and bone mass than midcourt players within both the senior and under-21 age groups. In comparison, senior players had greater lean mass, total BMD, and BMC than under-21 players. When scaled for height, senior goal circle and midcourters retained significantly greater lean mass compared to under-21 players. These differences between age groups, particularly the greater lean mass at the senior level, highlight the need for continued physical development in the under-21 squad before progressing to a senior squad. The findings also highlight the inter-individual variability in body composition within each squad. Across the three-season study, group-level changes in body composition and bone outcomes were minimal. Still, individual trends varied within each group, highlighting the importance of an individualised approach to body composition management in practice.

### Comparison between senior and under-21 positional groups

To the authors’ knowledge, this is the first study to investigate body composition and bone outcomes in NSL players using DEXA. Data from 47 netball players (23 senior and 24 under-21) across three seasons provide preliminary sport- and positional-specific comparative values for practitioners to guide physical preparation and development goals (Table 2). However, Figure 1 demonstrates the inter-individual variability in the body composition and bone outcomes within squads and positions, indicating the need for individualised consideration. Positional differences align with previous research in a professional SSN team^[9]^ and reflect the physical and technical requirements of each role.^[13,20]^ For example, midcourt players (e.g., centres) complete ~491 movement events per game compared to ~361 by a goal circle player (e.g., goal shooter) in the NSL,^[13]^ with elite teams often favouring a tall, holding goal shooter.^[20]^ Positional differences were more pronounced in senior than under-21 players (Table 2), suggesting greater positional specialisation at the senior level and ongoing development in younger players. Under-21 players had lower lean mass, bone mass, BMD, and BMC than the seniors, which is likely due to age, as noted by Simpson et al.^[10]^ found no differences in lean mass or BMD between elite and sub-elite senior players. The observed differences in body composition and bone outcomes highlight the developmental gap between age groups, emphasising the need for structured training and nutrition programmes to optimise physical development and long-term health.

When comparing senior NSL players to previously reported SSN values, the average total and scaled lean body mass appear similar, but NSL players have a higher fat mass percentage (NSL: 24.6 – 26.5% *vs.* SSN: 18.2 – 24.5%).^[9,10]^ While greater lean mass is linked to improved strength and power (e.g., jump height),^[21]^ the influence of greater fat mass on netball performance is unknown, but has been found to negatively impact power-to-weight ratio and intermittent-fitness test performance in rugby players.^[22]^ However, the health implications of, and achieving, low fat mass should be considered, particularly given the existing concerns of RED-s in NSL players.^[3]^ The observed difference from previous research ^[9,10]^ may reflect the NSL squad’s semi-professional status, where balancing work and training commitments can challenge nutrition and training optimisation and consistency. An increase in professionalism in the sport and its talent pathways could help support this through the implementation of more effective performance support.

Whole body bone health outcomes (BMD, BMC, and scaled bone mass) in NSL players were comparable to SSN values,^[9]^ whilst site-specific (i.e., hip and spine) measures were higher than reported in university and club level netball players.^[6]^ This aligns with research showing higher bone density in athletes compared to non-athletes, likely due to greater lean mass and the use of loaded exercises, and reinforces the need for sport-specific reference data (i.e., z-scores).^[7]^ This is the first study to consider site-specific bone health outcome measures in elite netball players, highlighting the within-group variability in values (Figure 1) and the need for caution when interpreting mean values alone. Yet, despite the spread of data, differences are present between the seniors and under-21s (Table 2). This may reflect continued growth and maturation, alongside greater exposure to elite netball training and match-play, reinforcing the need to prioritise continued physical development in younger netball players to enhance bone mass. The influence of menstrual dysfunction should also be considered, with research indicating that adolescent load-bearing females with menstrual dysfunction have higher stress fracture rates compared to eumenorrheic athletes (32% *vs.* 6%) ^[23]^ and the reported prevalence of menstrual and secondary amenorrhea in top-level student netball players is higher than reported in the general population.^[4]^ As such, given the lower bone mass, BMD and BMC within the under-21 age group, practitioners and coaches should also focus on menstrual health and adequate energy availability to support bone accrual and optimise bone health in pathway players.

### Within-season changes

Minimal group-level changes in body composition and bone outcomes were observed in the senior squad over the course of a season (Figures 2 and 3). This finding aligns with the observations of Hogarth and colleagues^[9]^ during the in-season period of an SSN team; however, they noted *small* increases in lean mass during the pre-season period. The maintenance of lean mass during the season suggests that sufficient training stimulus and/or energy balance are achieved, despite fixture congestion, travel demands, and reduced strength training (1–2 sessions per week). This can be seen as a favourable consideration, given the declines observed in some sports.^[24]^ Seasonal BMD changes have been reported in other team sports (e.g., soccer^[14]^), but the higher pre-season BMD in the senior netball players in the current study suggests that they may have reached a level of bone accrual that remains stable throughout the season. However, given the differences in body composition outcomes between senior and under-21 players, seasonal changes in lean and bone mass would be expected in the younger athletes. The lack of group-level changes observed could be due to the training demands and associated energy requirements. At the senior NSL level players require an average of 3250 kcal per day to achieve energy balance,^[2]^ but are also reported to be weight, body image and food conscious.^[3]^ Therefore, netball players do not consistently achieve the energy surplus required to build substantial lean mass. Importantly, individual variability in body composition and bone outcomes (Figure 1) highlights the need for personalised body composition management, particularly in semi-professional and developmental environments where competing demands vary between players.

### Strengths and limitations

This study provides the first detailed analysis of body composition and bone health outcomes in senior and under-21 NSL players, offering valuable comparative data to guide training and nutrition strategies for optimised body composition management. However, the absence of performance and injury data limits direct assessment of their impact on athletic performance and injury risk. In contrast, limited training load data, particularly at the under-21 level, restricts understanding of training stimulus effects. Future research should integrate detailed training load metrics and performance measures to clarify these relationships and understand how changes in lean and fat mass influence netball performance. Although conducted over three years, squad changes and data from a single NSL team limit generalisability. Expanding research across multiple teams and elite competitions (e.g., Telkom Netball League, ANZ Premiership) would enhance applicability. Despite these limitations, this study establishes a foundation for age- and position-specific monitoring of netball players, reinforcing the need for personalised support in semi-professional and developmental netball settings.

## Conclusion

In conclusion, differences exist in body composition and bone outcomes between age groups (seniors and under-21) and positional groups within an NSL franchise. Senior NSL players, particularly those in goal circle positions, exhibit superior stature, body mass, lean mass, and bone density compared to midcourters and under-21 players. These disparities likely reflect the specific demands of their positions, the effects of maturation, and the differences in training environments. The lack of significant differences in fat mass percentage among the groups suggests that the increased mass observed in senior players is primarily due to greater lean and bone mass rather than adiposity. The comparative data serve as a valuable reference for optimising training and development strategies in elite netball. The minimal changes observed in body composition and bone health outcomes at both levels of competition indicate that sufficient training stimulus and energy balance are maintained around competition to preserve lean mass. However, at the under-21 level, where physical development should be a priority, this could be considered insufficient. Further support and consideration for the training environment (e.g., training and nutrition interventions) should be provided at the under-21 level. Importantly, the individual variability within each group reinforces the need for an individualised approach to body composition management in practice. Future research should consider the impact of body composition and bone health changes on performance outcomes, health and injury in netball.

## Supplementary Information



## Figures and Tables

**Table 1:** General overview of weekly training content for a) the senior and b) under-21 Netball Super League squads

Period	Session type	Frequency (n)		Duration (minutes)	

		Senior	Under-21	Senior	Under-21

**Pre-season 1** (October – November)	Court sessions	4	2 to 4	61.9 ± 41.1	90 – 120
	Gym	3	2	59.7 ± 11.4	45 – 60
	Matches	0	1 to 2	60*	60*

**Pre-season 2 (senior) / in-season 1 (under-21)** (December – January)	Court sessions	4	2 to 4	89.1 ± 39.6	90 – 120
	Gym	3	2	60.0 ± 10.9	45–60
	Matches (friendlies)	1	1 to 3	60*	60*

**In season** (February – June)	Court sessions	2 to 3	2 to 4	82.3 ± 37.6	90 – 120
	Gym	2	2	62.6 ± 26.0	45 – 60
	Matches	1 to 2	1 to 3	60*	60*

* Standard netball format of 4 × 15-minute quarters

**Table 2:** Estimated means (95% confidence intervals) for body composition and bone outcome variables for Netball Super League (NSL) netball players by level and positional group

Variable	Senior		Under 21	
	Midcourter (n=10)	Gold Circle (n=13)	Midcourter (n=13)	Gold Circle (n=11)
Stature (cm)	174.1**## (172.4–175.7)	185.1** (183.7–186.6)	169.0 (167.5–170.5)	179.3 (177.7–180.8)
Body mass (kg)	71.0 (67.4–74.6)**##	85.4 (82.2–88.5)**	64.2 (61.0–67.3)#	69.3 (65.8–72.7)
Fat mass (kg)	16.9## (14.2–19.6)	24.2** (21.8–26.5)	16.8 (14.4–18.7)	17.7 (15.2–20.3)
Fat mass (%)	23.6# (21.4–25.9)	27.8 (25.6–30.0)	26.0 (23.9–28.2)	25.2 (23.0–27.5)
Scaled fat mass (kg·m^−2^)	5.6## (4.8–6.3)	7.1^*^ (6.4–7.8)	5.9 (5.2–6.6)	5.5 (4.8–6.2)
Lean mass (kg)	51.0**δ## (48.8–53.2)	57.7** (55.8–58.7)	44.6## (42.7–46.6)	48.6 (46.4–50.7)
Scaled lean mass (kg.m^−2^)	16.8^*^ (16.0–17.6)	17.1** (16.4–17.8)	15.6 (14.9–16.4)	15.1 (14.4–15.9)
Bone mass (kg)	4.0^*^## (2.9–3.0)	3.5** (3.4–3.6)	2.8 (2.6–2.9)	3.0 (2.8–3.1)
Scaled bone mass (kg·m^−2^)	1.02 (0.96–1.08)	1.03** (0.98–1.08)	0.97 (0.92–1.01)	0.92 (0.87–0.98)
Total body BMD (g·cm^−2^)	1.39^*^ (1.33–1.44)	1.42** (1.37–1.47)	1.29 (1.24–1.34)	1.29 (1.24–1.34)
Total body BMC (g)	3098**## (2941–3255)	3490** (3352–3628)	2756 (2618–2893)	2963 (2814–3113)
Total body BMD z-score	2.9 (2.2–3.5)	3.0 (2.2–3.5)	2.4 (1.7–3.0)	2.4 (1.8–3.0)
Total hip BMD (g·cm^−2^)	1.30 (1.22–1.37)	1.30 (1.23–1.36)	1.19 (1.13–1.26)	1.22 (1.14–1.29)
Total hip BMC (g)	43.0^*^# (40.1–46.0)	47.6** (45.0–50.1)	37.7 (35.1–40.3)	40.0 (37.2–42.8)
Total hip BMD z-score	2.1 (1.2–3.1)	2.3 (1.5–3.0)	1.6 (0.6–2.5)	1.8 (0.9–2.7)
Lumbar spine (L1 to L4) BMD (g·cm^−2^)	1.43 (1.34 – 1.51)	1.47^*^ (1.39 – 1.54)	1.41 (1.33 – 1.49)	1.34 (1.25 – 1.42)
Lumbar spine (L1 to L4) BMC (g)	85.15 (78.81 – 91.50)	93.9** (87.54 – 98.64)	79.24 (73.67 – 84.81)	78.26 (72.20 – 84.31)
Lumbar spine (L1–L4) BMD z-score	1.63 (0.75 – 2.51)	2.33^*^ (1.61 – 3.05)	2.17 (1.28 – 3.05)	1.17 (0.35 – 1.99)

** p<0.05 vs under 21.

** p<0.01 vs under 21

# p< 0.05 vs goal circle.

## p<0.01 vs goal circle.

BMD, bone mineral density; BMC, bone mineral content

## References

[1] Whitehead S Weakley J Cormack S The applied sports science and medicine of netball: a systematic scoping review Sports Med 2021 51 1715 31 10.1007/s40279-021-01461-6 34086257 PMC8310515

[2] Costello N Jones B Roe S Daily energy expenditure and water turnover in female netball players from the Netball Super League: A doubly labeled water observation study Eur J of Sport Sci 2024 24 1130 42 10.1002/ejsc.12160 39049758 PMC11295086

[3] O’Donnell J White C Dobbin N Perspectives on relative energy deficiency in sport (RED-S): A qualitative case study of athletes, coaches and medical professionals from a super league netball club PloS One 2023 18 e0285040 10.1371/journal.pone.0285040 37134124 PMC10155971

[4] Havemann L Lange ZD Pieterse K Wright HH Disordered eating and menstrual patterns in female university netball players S Afr J Sports Med 2011 23 68 72 [10.17159/2078-516X/2011/v23i3a328]

[5] Sale C Elliott-Sale KJ Nutrition and athlete bone health Sports Med 2019 49 139 51 10.1007/s40279-019-01161-2 31696454 PMC6901417

[6] Egan E Reilly T Giacomoni M Redmond L Turner C Bone mineral density among female sports participants Bone 2006 38 227 33 10.1016/j.bone.2005.08.024 16257279

[7] Mountjoy M Sundgot-Borgen J Burke L The IOC consensus statement: beyond the female athlete triad—Relative Energy Deficiency in Sport (RED-S) Br J Sports Med 2014 48 491 7 10.1136/bjsports-2014-093502 24620037

[8] Hancock Jonvik KL Torstveit MK Sundgot-Borgen J Mathisen TF Do we need to change the guideline values for determining low bone mineral density in athletes? J Appl Physiol 2022 132 1320 2 10.1152/japplphysiol.00851.2021 35060767 PMC9126212

[9] Hogarth L Farley A McKenzie M Burkett B McKean M Body composition in professional female netball players within and between seasons: a cohort study BMC Sports Sci, Med Rehab 2021 13 10.1186/s13102-021-00287-z 34088361 PMC8176725

[10] Simpson M Jenkins D Leveritt M Kelly V Physical profiles of elite, sub-elite, regional and age-group netballers J Sports Sci 2018 37 1212 9 [10.1080/02640414.2018.1553269 30558478

[11] Kohrt WM Barry DW Schwartz RS Muscle forces or gravity: What predominates mechanical loading on bone? Med Sci Sports Exerc 2009 41 2050 5 [10.1249/MSS.0b013e3181a8c717] 19812511 PMC3037021

[12] Brooks ER Benson AC Fox AS Bruce LM Physical movement demands of training and matches across a full competition cycle in elite netball Appl Sci 2020 10 7689 [10.3390/app10217689]

[13] Mackay L Jones B Owen C Movement characteristics of international and elite domestic netball players during matchplay Int J Perf Anal Sport 2025 1 144 161 [10.1080/24748668.2024.2303893]

[14] Varley I Ward M Thorpe C External training load is associated with adaptation in bone and body composition over the course of a season in elite male footballers Bone Rep 2023 18 101643 10.1016/j.bonr.2022.101643 36531121 PMC9747571

[15] Agostinete RR Vlachopoulos D Werneck AO Bone accrual over 18 months of participation in different loading sports during adolescence Arch Osteoporos 2020 15 64 10.1007/s11657-020-00727-2 32335776

[16] Esparza-Ros F Vaquero-Cristóbal R Anthropometry Fundamentals of Application and Interpretation Switzerland, Cham Springer Nature 2025

[17] Krueger D Tanner SB Szalat A DXA Reporting Updates: 2023 official positions of the International Society for Clinical Densitometry Journal of Clinical Densitometry 2024 27 101437 10.1016/j.jocd.2023.101437 38011777

[18] Scantlebury S Costello N Owen C Longitudinal changes in anthropometric, physiological, and physical qualities of international women’s rugby league players PloS One 2024 19 5 e0298709 [10.1371/journal.pone.0298709 38743656 PMC11093382

[19] Douma JC Weedon JT Analysing continuous proportions in ecology and evolution: A practical introduction to beta and Dirichlet regression Methods Ecol Evol 2019 10 1412 30 [10.1111/2041-210X.13234]

[20] Bruce L Brooks ER Woods CT Team and seasonal performance indicator evolution in the ANZ Championship netball league J Sports Sci 2018 36 2771 7 [10.1080/02640414.2018.1473099] 29745299

[21] Stephenson ML Smith DT Heinbaugh EM Total and lower extremity lean mass percentage positively correlates with jump performance J Strength Cond Res 2015 29 2167 10.1519/JSC.0000000000000851 25627641

[22] Darrall-Jones J Roe G Carney S The Effect of Body Mass on the 30-15 Intermittent Fitness Test in Rugby Union Players Int J Sports Physiol Perform 2016 11 400 3 10.1123/ijspp.2015-0231 26217047

[23] Ackerman KE Cano Sokoloff N Maffazioli GDN Clarke H Lee H Misra M Fractures in relation to menstrual status and bone parameters in young athletes Med Sci Sports Exerc 2015 47 1577 86 10.1249/MSS.0000000000000574 25397605 PMC4430468

[24] Georgeson EC Weeks BK McLellan C Beck BR Seasonal change in bone, muscle and fat in professional rugby league players and its relationship to injury: a cohort study BMJ Open 2012 2 6 e001400 [10.1136/bmjopen-2012-001400 23135539 PMC3532969

