# Supplementary material for: Body composition and bone health outcomes across positional groups in Netball Super League (NSL) senior and under-21 players; a multi-year cohort study
Source: S Afr J Sports Med. 2025 Dec 15;37(1):v37i1a22881. doi: 10.17159/2078-516X/2025/v37i1a22881 (PMC12726998; doi:10.17159/2078-516X/2025/v37i1a22881)
Supplement: Supplementary file 1 [file 2078-516X-37-v37i1a22881-s001.pdf]

Body composition and bone health outcomes across positional groups in Netball Super League (NSL) senior and under-21 players; a multi-year cohort study

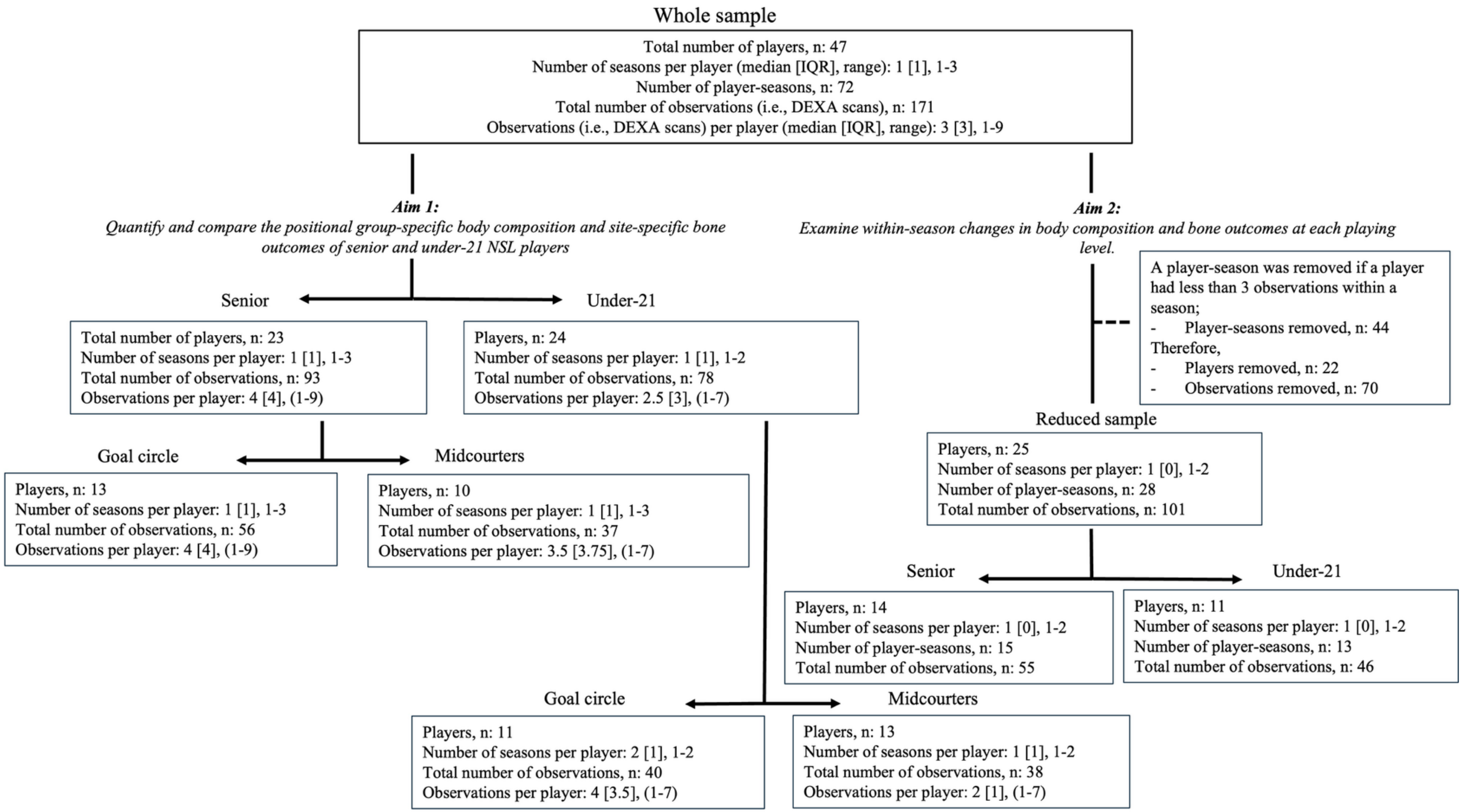

Supplementary Fig. 1. *A breakdown of the whole sample and sample used to address each aim is shown in the flow chart below. Data were collected over three seasons, therefore the median (interquartile range) and range of the number of seasons per player are provided. The number of 'player-seasons' refers to the total number of seasons across the whole dataset (i.e., each player-season represents one players' data in one season). 'Observations' are referring to DEXA scans, with total and observations per player provided. For aim one, the whole sample was used. The flow chart indicates how the data were split into subgroups, firstly by age-group then by positional group and the breakdown of the sample in each group. The statistical analysis conducted takes into account the repeated player observations. For aim two, a reduced sample was used. A player-season was only included if it had three or more observations. In some instances, removing a player-season resulted in the removal of a player from the dataset entirely. Whilst in other instances, a player may have remained in the dataset due to having three or more observations in another season. The flow chart shows the reduced sample and the split into age-groups.*

Supplementary Table 1. Positional and age group comparisons for body composition from linear mixed effect models.

Variable	Positional comparison				Level comparison			
	Senior (goal circle vs. midcourt)		Under-21 (goal circle vs. midcourt)		Goal circle (senior vs. under-21)		Midcourt (senior vs. under-21)	
	**P value**	**ES (95% CI)**	**P value**	**ES (95% CI)**	**P value**	**ES (95% CI)**	**P value**	**ES (95% CI)**
Stature (cm)	**<0.01**	4.22 (3.38–5.06)	**<0.01**	3.93 (3.11–4.74)	**<0.01**	-2.24 (-3.05- -1.42)	**<0.01**	-1.94 (-2.78- -1.10)
Total body mass (kg)	**<0.01**	1.82 (1.21–2.43)	**0.03**	0.65 (0.06–1.24)	**<0.01**	-2.04 (-2.63–2.73)	**0.01**	-0.87 (-1.47 – -0.26)
Fat mass (kg)	**<0.01**	1.22 (0.61-1.82)	0.59	0.16 (-0.43-0.75)	**<0.01**	-1.08 (-1.67- -0.49)	0.95	0.02 (-0.63-0.59)
Scaled fat mass (kg·m-2)	**<0.01**	0.89 (0.30-1.47)	0.42	-0.23 (-0.80-0.34)	**<0.01**	-0.93 (-1.50- -0.36)	0.52	0.19 (-0.40-0.78)
Lean mass (kg)	**<0.01**	1.44 (0.81–2.07)	**<0.01**	0.84 (0.23-1.46)	**<0.01**	-1.97 (-2.58- -1.35)	**<0.01**	-1.37 (-2.01- -0.74)
Scaled lean mass (kg·m-2)	0.59	0.18 (-0.48-0.84)	0.34	-0.31 (-0.95-0.34)	**<0.01**	-1.17 (-1.82- -0.53)	**0.04**	-0.69 (-1.35- -0.03)
Bone mass (kg)	**<0.01**	1.45 (0.70-2.23)	0.05	0.77 (0.016-1.52)	**<0.01**	-1.95 (-2.71- -1.20)	**<0.01**	-1.27 (-2.04- -0.50)
Scaled bone mass (kg·m-2)	0.70	0.15 (-0.63-0.93)	0.25	-0.44 (-1.20-0.32)	**<0.01**	-1.15 (-1.92- -0.39)	0.15	-0.56 (-1.35-0.22)
Total body BMD (g·cm-2)	0.33	0.33 (-0.35–1.02)	0.94	0.02 (-0.64–0.69)	**<0.01**	-1.17 (-1.83 - -0.50)	**0.02**	-0.85 (-1.53 - -0.17)
Total body BMC (g)	**<0.01**	1.45 (0.68–2.23)	0.05	0.77 (0.12 – 1.52)	**<0.01**	-1.95 (-2.71 - -1.20)	**<0.01**	-1.27 (-2.04- -0.46)
Total body BMD z-score	0.76	0.10 (-0.49–0.70)	0.98	-0.04 (-0.63-0.55)	0.13	-0.46 (-1.04-0.11)	0.26	-0.32 (-0.93-0.28)
Total hip BMD (g·cm-2)	0.96	-0.02 (-0.79-0.76)	0.65	0.17 (-0.58-0.93)	0.12	-0.59 (-1.35-0.16)	0.05	-0.78 (--1.56- -0.01)
Total hip BMC (g)	**0.02**	0.88 (0.12-1.64)	0.22	0.48 (-0.28-1.20)	**<0.01**	-1.47 (-2.21- -0.73)	**0.01**	-1.04 (-1.81- -0.28)
Total hip z-score	0.83	0.19 (-0.84–1.12)	0.71	0.11 (-0.87–1.08)	0.42	0.38 (-0.56–1.31)	0.39	0.46 (-0.61–1.53)
L1-L4 BMD (g·cm-2)	0.51	0.2 (-0.46-0.93)	0.19	-0.44 (-1.120.24)	**0.03**	-0.78 (-1.45- -0.10)	0.19	-0.10 (-0.80-0.59)
L1-L4 BMC (g)	0.06	0.61 (-0.04-1.26)	0.81	-0.08 (-0.71-0.56)	**<0.01**	-1.14 (-1.77- -0.51)	0.17	-0.45 (-1.10-0.19)
L1-L4 BMD z-score	0.22	0.58 (-0.35-1.51)	0.10	-0.81 (-1.80-0.17)	**0.04**	0.96 (0.06-1.85)	0.39	-0.44 (-1.46-0.59)
	P value	OR (95%CI)	P value	OR (95%CI)	P value	OR (95%CI)	P value	OR (95%CI)
Fat mass (%)	**0.01**	1.25 (1.05-1.47)	0.61	0.96 (0.82-1.13)	0.11	1.14 (0.97-1.34)	0.13	0.88 (0.74-1.04)

CI = confidence intervals, OR = odds ratio, BMD = bone mineral density, BMC = bone mineral content.
Bold dictates p<0.05

= *trivial ES*	= *small* ES	= *moderate* ES	= *large* ES	= *very large* ES	= *extremely large* ES
